# Association between serum sodium levels within 24 h of admission and all-cause mortality in critically ill patients with non-traumatic subarachnoid hemorrhage: a retrospective analysis of the MIMIC-IV database

**DOI:** 10.3389/fneur.2023.1234080

**Published:** 2023-09-15

**Authors:** Junjie Liu, Jianmin Li, Qiuhua Zhang, Liang Wang, Yichao Wang, Jingxi Zhang, Junwei Zhang

**Affiliations:** ^1^College of Clinical Medicine, North China University of Science and Technology, Tangshan, China; ^2^Department of Critical Care Medicine, The Affiliated Hospital North China University of Science and Technology, Tangshan, China

**Keywords:** subarachnoid hemorrhage, admission serum sodium, in-hospital mortality, ICU mortality, MIMIC-IV database

## Abstract

**Objective:**

The study aimed to evaluate the relationship between serum sodium and mortality in critically ill patients with non-traumatic subarachnoid hemorrhage.

**Methods:**

This is a retrospective investigation of critically ill non-traumatic patients with subarachnoid hemorrhage (SAH) utilizing the MIMIC-IV database. We collected the serum sodium levels at admission and determined the all-cause death rates for the ICU and hospital. We employed a multivariate Cox proportional hazard regression model and Kaplan–Meier survival curve analysis to ascertain the relationship between serum sodium and all-cause mortality. In order to evaluate the consistency of correlations, interaction and subgroup analyses were also conducted.

**Results:**

A total of 864 patients with non-traumatic SAH were included in this study. All-cause mortality in the ICU and hospital was 32.6% (282/864) and 19.2% (166/864), respectively. Sodium levels at ICU admission showed a statistically significant J-shaped non-linear relationship with ICU and hospital mortality (non-linear *P*-value < 0.05, total *P*-value < 0.001) with an inflection point of ~141 mmol/L, suggesting that mortality was higher than normal serum sodium levels in hypernatremic patients. Multivariate analysis after adjusting for potential confounders showed that high serum sodium levels (≥145 mmol/L) were associated with an increased risk of all-cause mortality in the ICU and hospital compared with normal serum sodium levels (135–145 mmol/L), [hazard ratio (HR) = 1.47, 95% CI: 1.07–2.01, *P* = 0.017] and (HR = 2.26, 95% CI:1.54–3.32, *P* < 0.001). Similarly, Kaplan–Meier (K-M) survival curves showed lower survival in patients with high serum sodium levels. Stratified analysis further showed that the association between higher serum sodium levels and hospital all-cause mortality was stronger in patients aged < 60 years with a hospital stay of <7 days.

**Conclusion:**

High serum sodium levels upon ICU admission are related to higher ICU and hospital all-cause mortality in patients with non-traumatic SAH. A new reference is offered for control strategies to correct serum sodium levels.

## 1. Introduction

Subarachnoid hemorrhage (SAH) is a severe subtype of hemorrhagic stroke, accounting for 5% of stroke patients (1) and a mortality rate of up to 35% within 30 days (2). In China, the in-hospital mortality rate of SAH is ~3.7% (3); non-traumatic SAH, with ruptured intracranial aneurysms, is the primary cause in most cases, and many patients with SAH require admission to the intensive care unit (ICU) for a variety of reasons, about 30% patients with SAH are fatal, and at least 20% of survivors do not regain functional independence (4, 5). Physical disability and psychological impairment (6). Despite extensive clinical studies and maximal treatment, patients with SAH continue to exhibit discouraging clinical outcomes (7). Because of the high mortality and low cure rates, it is imperative to identify predictors of short-term or long-term prognostic outcomes.

In recent decades, the burden of stroke has been steadily increasing in China, with a rising incidence of subarachnoid hemorrhage (SAH), while the treatment outcomes remain suboptimal (8). Therefore, it is necessary to explore predictive indicators for SAH mortality. Currently, there is a lack of relevant SAH databases in China; hence, we selected SAH patients from the MIMIC database in the United States as our study subjects. Sodium imbalance is the most common electrolyte disturbance among critically ill SAH patients, leading to potential adverse outcomes (9). Sodium ion is the predominant cation in the extracellular fluid, such as blood. It plays a crucial role in maintaining appropriate osmotic pressure, cellular physiology, extracellular fluid volume, and acid–base balance (10). Activation of the renin–angiotensin–aldosterone system (RAAS), antidiuretic hormone release, and sympathetic nervous system stimulation are likely to be the primary factors contributing to poor prognosis in SAH (11). Currently, there is limited research describing the characteristics of sodium fluctuations and their potential prognostic implications in critically ill non-traumatic SAH patients in the intensive care unit (ICU).

We propose to extract patients with non-traumatic subarachnoid hemorrhage from the MIMIC IV database and conduct a retrospective cohort study to explore the causal relationship between their serum sodium and in-hospital mortality to better manage electrolytes and ultimately improve clinical prognosis.

## 2. Materials and methods

### 2.1. Study population

The cohort of critically ill patients with non-traumatic SAH was drawn from the Intensive Care IV (MIMIC-IV; version 2.0) database, which comprises extensive clinical data from 60,000 ICU patients at Beth Israel Deaconess Medical Center between 2008 and 2019. The use of the database was authorized by the MIT and Beth Israel Deaconess Medical Center (BIDMC, Boston, Massachusetts, USA) institutional review boards. Junjie Liu, one of the authors, completed the NIH online course “Protecting Human Research Participants” (Record ID: 52698592) and was allowed database access for data extraction. All data were de-identified in order to protect patient privacy. Therefore, the Beth Israel Deaconess Medical Center Ethics Committee waived informed consent. The study was presented in compliance with the Statement for Strengthening Reports of Observational Studies in Epidemiology (STROBE) and the Helsinki Declaration standards.

SAH was diagnosed using the International Classification of Diseases Ninth (ICD-9) and Tenth Revisions (ICD-10).

Patients who met all of the criteria were selected for analysis: (1) first ICU admission; (2) age > 18 years; and (3) documented serum sodium test completed within 24 h of admission. The exclusion criteria were as follows: (1) ICU patients with a length of stay <24 h; (2) clear trauma etiology; (3) ICU readmission; and (4) missing data > 5%.

### 2.2. Data acquisition

On the 1st day of ICU admission, the following variables were retrieved from the aforementioned database: (1) demographic variables: gender, age, race, and insurance; (2) vital signs: temperature, heart rate, blood pressure, and oxygen saturation; (3) coexisting diseases: myocardial infarction, congestive heart failure, peripheral vascular disease, cerebrovascular disease, chronic lung disease, mild liver disease, diabetes mellitus, and tumors; (4) within the first 24 h after ICU admission, laboratory indicators such as glucose, white blood cell count (WBC), hemoglobin, platelets, potassium, chloride, blood urea nitrogen (BUN), blood creatinine, bicarbonate, anion gap, international normalized ratio (INR), prothrombin time (PT), and activated partial thromboplastin time (APTT) were detected. The mean value was utilized if a variable was discovered more than once in the first 24 h; (5) Glasgow Coma Scale (GCS), Simplified Acute Physiology Score II (SAPS II), and Acute Physiology Score III (APS III) were used to evaluate severity at the time of admission; and (6) ICU stay duration, length of stay, ICU death, and in-hospital death were all reported.

### 2.3. Endpoints

Endpoints included ICU death, in-hospital death, ICU duration of stay, and overall length of stay.

### 2.4. Statistics

The continuous variables were represented as mean, SD, or median (interquartile spacing). Depending on the distribution's normality, the independent samples *t*-test or Mann–Whitney *U*-test was performed. Moreover, non-normally distributed variables were represented as the median interquartile range (IQR) and compared between groups using the Wilcoxon rank sum test. The hypothesis for categorical variables was tested using the number of cases (%) and the chi-square test (or Fisher's exact methods).

Serum sodium concentrations were classified into three groups: ≤ 135, 135–145, and ≥145. The association between serum sodium levels and ICU or hospitalization outcomes was investigated.

The association between serum sodium and ICU mortality and in-hospital mortality was investigated using univariate and multivariate logistic regression models. Three models were used: model 1 was unadjusted; model 2 was adjusted for age, sex, and race; and model 3 was adjusted for systolic blood pressure (SBP), diastolic blood pressure (DBP), respiratory rate, heart rate, GCS, SAPS II, APS III, diabetes, renal failure, chronic lung disease, leukocytes, platelets, hemoglobin, glucose, BUN, creatinine, bicarbonate, and chloride.

Furthermore, we performed curve fitting to determine the linear connection between serum sodium and ICU or in-hospital mortality. We employed hierarchical linear regression models and likelihood ratio tests to determine alterations: age (<60 or ≥60 years), GCS score (<8 or ≥8), SAPS II score (<30 or ≥30), and length of ICU stay (<7 or ≥7 days). For all statistical studies, the R software package (http://www.R-project.org, The R Foundation) and Free Statistics software version 1.7 were used. A *P*-value of <0.05 was used to determine statistical significance.

## 3. Results

### 3.1. Baseline characteristics

**Figure 1:**
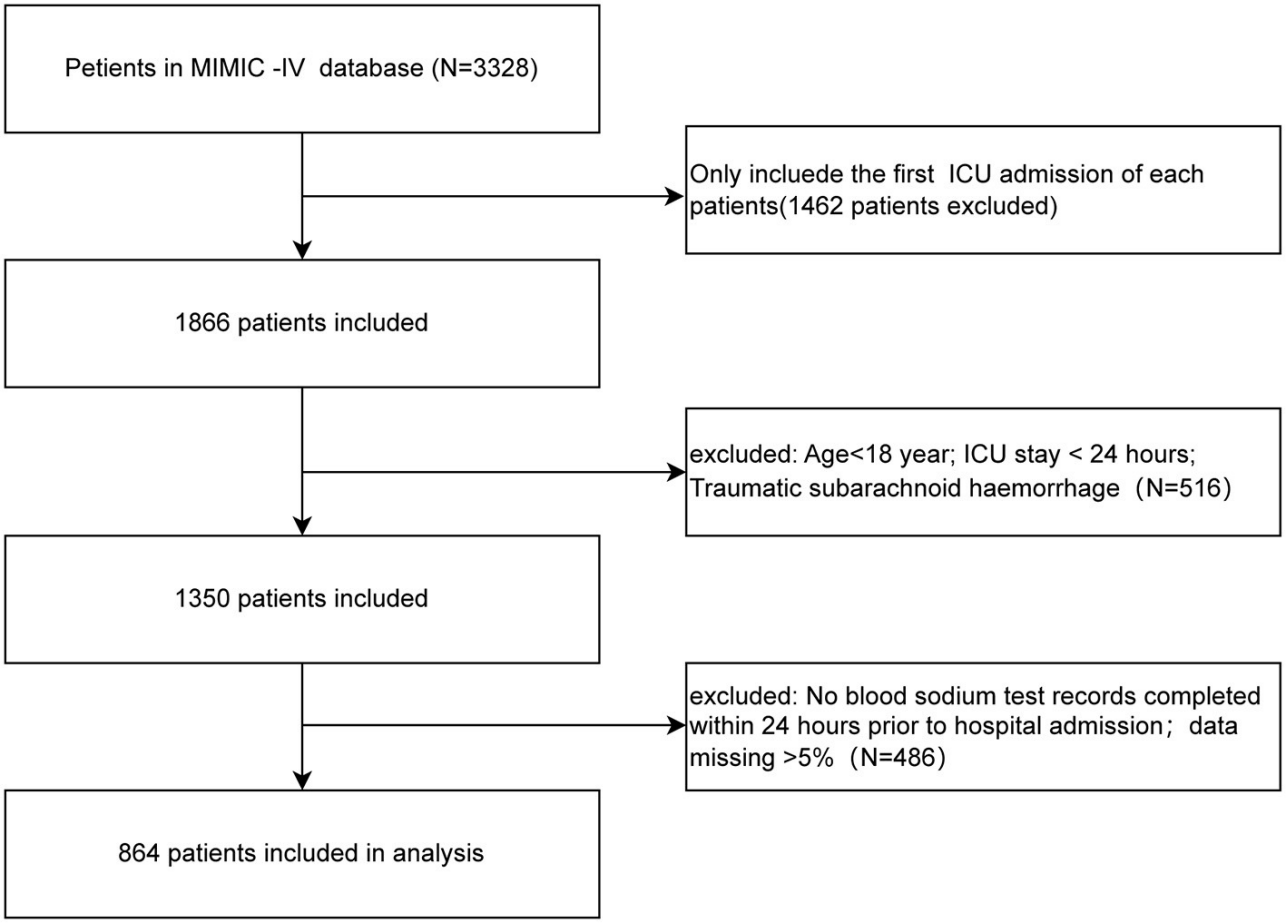
The flow chart of this study.

Figure 1 shows the enrollment flowchart based on the inclusion and exclusion criteria.

Table 1 shows that a total of 864 individuals with non-traumatic SAH were included, with 398 of them being males. The mean age was 64.0 ± 17.1 years. Among these 864 patients, 698 survived and 166 died in the hospital, while 582 survived and 282 died in the ICU, yielding an overall in-ICU mortality rate of 32.6% and an in-hospital mortality rate of 19.2%. There were significant differences in heart rate, blood pressure, SAPS II score, and APS III score between the hyponatremia and hypernatremia groups compared to the normonatremia group (*P*-value < 0.05), and regarding laboratory data chloride, bicarbonate, and anion gap were significantly different (*P*-value < 0.05). In addition, the results revealed that the total length of stay was shorter in patients with high serum sodium levels (≥145 mmol/L), while the length of ICU stay was not significantly different compared with other groups; ICU mortality and in-hospital mortality were the lowest in patients with normal serum sodium levels (≥135, <145 mmol/L), while ICU mortality and in-hospital mortality were the highest in patients with high serum sodium levels (≥145 mmol/L) (*P*-value < 0.05).

**Table 1:** Baseline characteristics related to in-hospital mortality.

	**Serum sodium (mmol/L)**				
	**Total (*****n*** = **864)**	**Tertile (**<**135) (*****n*** = **39)**	**Tertile (**≥**135**,<**145) (*****n*** = **691)**	**Tertile (**≥**145) (*****n*** = **134)**	***P*** **-value**
Age, year	64.0 ± 17.1	67.5 ± 16.3	64.3 ± 17.1	61.5 ± 17.0	0.094
**Gender**, ***n*** **(%)**					0.441
Female	466 (53.9)	18 (46.2)	371 (53.7)	77 (57.5)	
Male	398 (46.1)	21 (53.8)	320 (46.3)	57 (42.5)	
**Race**, ***n*** **(%)**					0.029
White	551 (63.8)	24 (61.5)	459 (66.4)	68 (50.7)	
Asian	28 (3.2)	0 (0)	23 (3.3)	5 (3.7)	
Black	52 (6.0)	3 (7.7)	40 (5.8)	9 (6.7)	
Other	233 (27.0)	12 (30.8)	169 (24.5)	52 (38.8)	
**Insurance**, ***n*** **(%)**					0.173
Medicaid	57 (6.6)	2 (5.1)	49 (7.1)	6 (4.5)	
Medicare	317 (36.7)	19 (48.7)	257 (37.2)	41 (30.6)	
Other	490 (56.7)	18 (46.2)	385 (55.7)	87 (64.9)	
HR, beats/min	79.8 ± 14.2	83.0 ± 16.0	78.6 ± 13.4	85.0 ± 16.2	<0.001
SBP, mmHg	124.7 ± 14.3	129.8 ± 14.4	125.1 ± 14.3	121.2 ± 13.5	0.001
DBP, mmHg	63.9 ± 9.6	67.2 ± 11.1	63.4 ± 9.5	65.5 ± 9.1	0.006
RR, beats/min	25.7 ± 5.7	26.3 ± 5.5	25.5 ± 5.7	26.3 ± 5.6	0.304
Temperature, °C	37.6 ± 0.7	37.5 ± 0.5	37.5 ± 0.7	37.7 ± 0.9	0.006
Spo2, mean ± SD	91.8 ± 10.1	92.0 ± 7.6	92.2 ± 8.4	90.0 ± 16.6	0.068
Myocardial infarct	60 (6.9)	3 (7.7)	51 (7.4)	6 (4.5)	0.477
Congestive heart failure	73 (8.4)	7 (17.9)	54 (7.8)	12 (9)	0.095
Peripheral vascular disease	77 (8.9)	6 (15.4)	57 (8.2)	14 (10.4)	0.211
Cerebrovascular disease	572 (66.2)	25 (64.1)	449 (65)	98 (73.1)	0.181
Chronic pulmonary disease	109 (12.6)	6 (15.4)	89 (12.9)	14 (10.4)	0.625
Mild liver disease	41 (4.7)	2 (5.1)	29 (4.2)	10 (7.5)	0.216
Diabetes	136 (15.7)	6 (15.4)	115 (16.6)	15 (11.2)	0.284
Metastatic solid tumor	15 (1.7)	1 (2.6)	11 (1.6)	3 (2.2)	0.465
Glucose, mg/dL	135.2 (116.6, 158.8)	125.5 (112.8, 144.2)	135.3 (116.6, 159.0)	140.2 (120.0, 160.6)	0.075
WBC, 10^9^/L	13.5 ± 6.4	11.8 ± 5.1	13.2 ± 6.3	15.0 ± 7.3	0.003
Hemoglobin, g/dL	11.6 ± 2.1	11.5 ± 1.8	11.7 ± 2.1	11.3 ± 2.2	0.147
platelets, 10^9^/L	202.6 ± 82.3	202.5 ± 95.2	203.9 ± 80.3	196.5 ± 88.7	0.638
Potassium, mmol/L	4.3 ± 0.7	4.3 ± 0.7	4.3 ± 0.8	4.3 ± 0.6	0.979
Chloride, mmol/L	107.1 ± 5.6	97.3 ± 4.2	106.3 ± 3.8	114.0 ± 6.8	<0.001
BUN, mg/dL	19.9 ± 12.7	19.5 ± 10.2	19.7 ± 12.7	21.0 ± 13.1	0.522
Creatinine, mg/dL	1.0 ± 0.8	1.1 ± 1.1	1.0 ± 0.8	1.1 ± 0.6	0.627
Bicarbonate, mmoL/L	24.8 ± 3.3	24.1 ± 2.6	24.7 ± 3.2	25.4 ± 4.0	0.03
Aniongap, mmoL/L	16.0 ± 3.4	16.8 ± 2.9	15.8 ± 3.3	17.0 ± 4.1	<0.001
INR	1.1 ± 0.2	1.1 ± 0.3	1.1 ± 0.2	1.1 ± 0.2	0.716
PT,s	14.4 ± 8.7	14.4 ± 6.5	14.4 ± 9.3	14.6 ± 5.8	0.982
APTT,s	28.5 (25.6, 33.5)	29.0 (26.1, 34.6)	28.4 (25.6, 32.9)	29.2 (25.4, 35.8)	0.430
GCS	13.9 ± 1.9	13.8 ± 2.0	13.9 ± 1.9	13.9 ± 2.1	0.93
SAPS II	33.4 ± 13.1	33.4 ± 12.6	31.9 ± 12.4	41.1 ± 14.3	<0.001
APS III	39.0 (28.0, 56.0)	41.0 (30.5, 61.5)	37.0 (27.0, 52.0)	51.0 (32.2, 74.5)	<0.001
Length of ICU stay, days	3.0 (1.0, 9.0)	5.0 (1.5, 9.5)	3.0 (1.0, 8.0)	4.0 (2.0, 11.0)	0.29
Length of hospital stay, days	8.0 (4.0, 15.0)	9.0 (6.0, 15.5)	9.0 (4.0, 14.0)	7.0 (2.0, 16.0)	0.038
ICU mortality, *n* (%)	282 (32.6)	12 (30.8)	201 (29.1)	69 (51.5)	<0.001
Hospital mortality, *n* (%)	166 (19.2)	8 (20.5)	105 (15.2)	53 (39.6)	<0.001

SAH, subarachnoid hemorrhage; SBP, systolic blood pressure; DBP, diastolic blood pressure; MBP, mean blood pressure; RR, respiratory rate; HR, heart rate; SpO2, percutaneous oxygen saturation; SAPS II, simplified acute physiology score II; GCS, Glasgow Coma Score; Chronic pulmonary disease, chronic obstructive pulmonary disease; WBC, white blood cell; BUN, blood urea nitrogen.

### 3.2. Association between serum sodium and all-cause mortality in non-traumatic SAH

**Figure 2:**
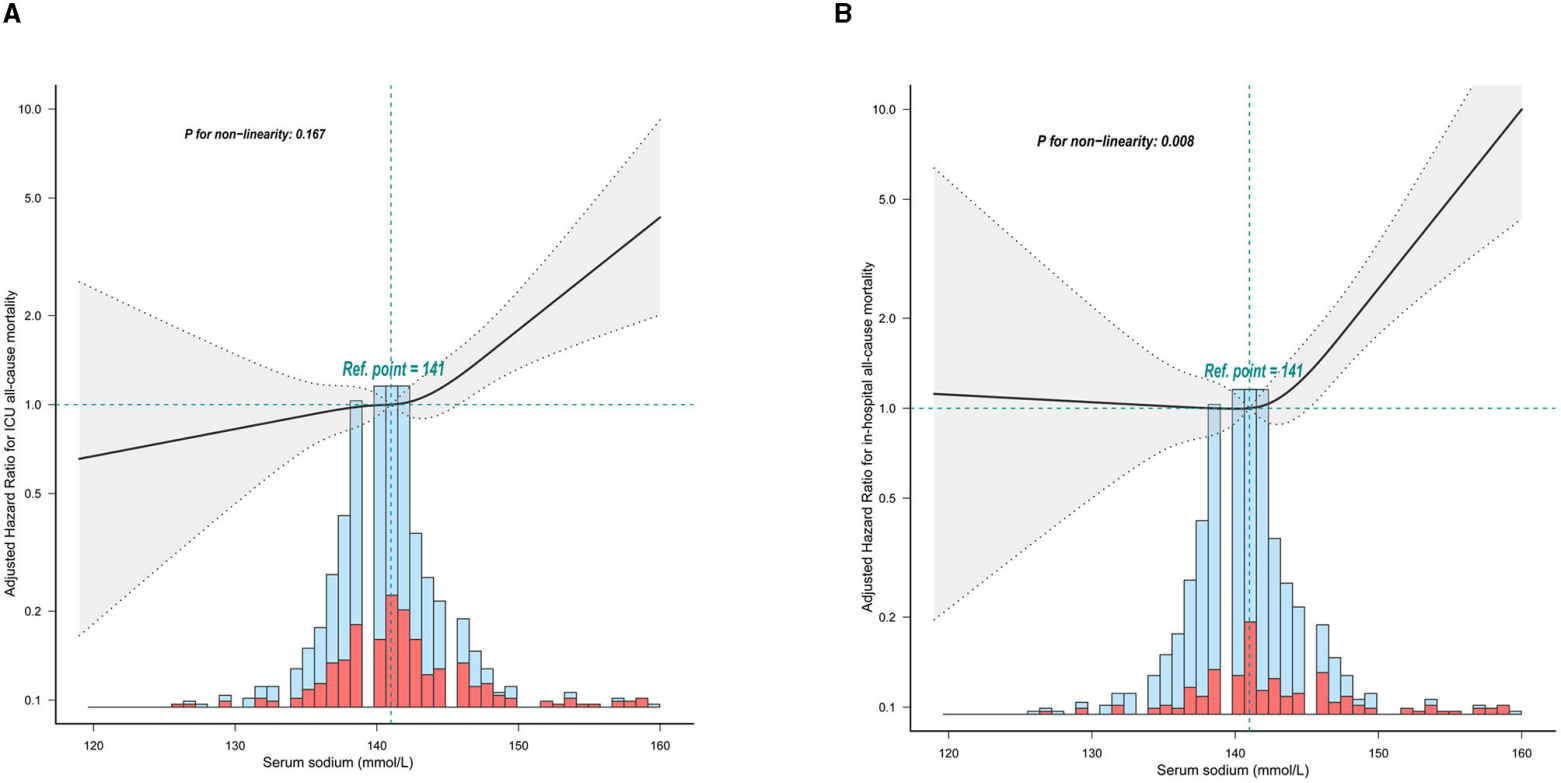
Relationship between serum sodium and ICU mortality and in-hospital mortality within 24 h of admission. The solid line represents the smoothed curve fit between the variables. The res dashed line represents the 95% confidence interval of the fit. **(A)** ICU all-cause mortality; **(B)** Hospital all-cause mortality. The solid red line represents the smooth curve fit between variables. Data were adjusted for age, sex, ethnicity, SBP, DBP, respiratory rate, GCS, diabetes, sepsis, renal failure, chronic pulmonary disease, vasopressor, embolization of aneurysm, clipping of aneurysm, WBC, platelet, hemoglobin, glucose, BUN, and creatinine.

In participants with non-traumatic SAH, a restricted three-sample analysis revealed a non-linear connection between serum sodium and ICU all-cause mortality, which was consistent with hospital all-cause mortality. In non-traumatic SAH, serum sodium levels (<141 mmol/L) were not significantly related with ICU and hospital all-cause mortality. As indicated in Figure 2 and Table 2, increasing levels of serum sodium (≥141 mmol/L) and increased ICU and hospital all-cause mortality were seen in non-traumatic SAH.

**Table 2:** Threshold analysis of serum sodium on ICU mortality and in-hospital mortality in patients with SAH using a two-segment regression model.

**Threshold of sodium, mol/L**	**HR**	**95% CI**	***P*-value**
**ICU all-cause mortality**			
<141	1.04	0.981, 1.103	0.1908
≥141	1.098	1.059, 1.139	<0.001
Likelihood ratio test			0.002
**Hospital all-cause mortality**			
<141	1.081	0.994, 1.175	0.0695
≥141	1.155	1.097, 1.215	<0.001
Likelihood ratio test			0.001

SAH, subarachnoid hemorrhage; HR, hazard ratio; CI, confidence interval. Adjusting factors included: age, sex and race, SBP, DBP, respiratory rate, heart rate, GCS, SAPS II, APS III, diabetes mellitus, renal failure, chronic lung disease, white blood cells, platelets, hemoglobin, glucose, BUN, creatinine, bicarbonate, and chloride.

As shown in Table 3, univariate Cox regression analysis revealed that age, systolic blood pressure, respiratory rate, oxygen saturation, vascular disease, hemoglobin, urea nitrogen, blood creatinine, anion gap, INR, PT, PTT, GCS, SAPS II, APS III, and serum sodium were all significantly associated with ICU and in-hospital mortality.

**Table 3:** Univariate cox analysis between serum sodium level and mortality.

	**ICU mortality**		**In-hospital mortality**	
**Variable**	**HR (95% CI)**	***P***	**HR (95% CI)**	***P***
Age	1.04 (1.03, 1.05)	<0.001	1.03 (1.02, 1.04)	<0.001
Gender: male vs. female	1.15 (0.91, 1.45)	0.241	0.98 (0.72, 1.33)	0.909
**Race**, ***n*** **(%)**				
White	1		1	
Asian	1.27 (0.67, 2.4)	0.466	1.81 (0.84, 3.92)	0.131
Black	1.05 (0.62, 1.79)	0.854	0.72 (0.32, 1.66)	0.443
Other	1.2 (0.93, 1.55)	0.163	1.91 (1.39, 2.63)	<0.001
**Insurance**				
Medicaid	1		1	
Medicare	4.61 (2.27, 9.4)	<0.001	0.82 (0.4, 1.66)	0.577
Other	1.8 (0.88, 3.69)	0.107	0.62 (0.29, 1.34)	0.222
HR, beats/min	1.01 (1.00, 1.01)	0.016	1.73 (0.81, 3.73)	0.159
SBP, mmHg	2.03 (1.87, 3.15)	0.015	3.04 (1.5, 6.13)	0.002
DBP, mmHg	0.99 (0.98, 1.01)	0.450	0.98 (0.77, 1.03)	0.561
RR, beats/min	1.04 (1.02, 1.06)	<0.001	3.85 (1.56, 9.5)	0.003
Temperature, °C	0.81 (0.68, 0.96)	0.014	1.86 (0.75, 4.61)	0.178
Spo2, Mean ± SD	0.97 (0.96, 0.98)	<0.001	1.02 (1, 1.03)	0.004
Myocardial infarct	1.07 (0.72, 1.61)	0.734	0.99 (0.98, 1.00)	0.699
Congestive heart failure	1.72 (1.23, 2.42)	0.002	0.98 (0.97, 1)	0.04
Peripheral vascular disease	0.59 (0.38, 0.93)	0.021	1.04 (1.01, 1.06)	0.002
Cerebrovascular disease	0.58 (0.44, 0.75)	<0.001	0.96 (0.76, 1.21)	0.741
Chronic pulmonary disease	1.26 (0.91, 1.74)	0.158	0.96 (0.96, 0.97)	<0.001
Mild liver disease	1.3 (0.81, 2.1)	0.279	1.3 (0.79, 2.15)	0.305
Diabetes	1.36 (1.01, 1.83)	0.04	1.5 (0.95, 2.37)	0.082
Metastatic solid tumor	2.84 (1.51, 5.34)	0.001	0.83 (0.48, 1.44)	0.512
Glucose, mg/dL	1 (0.99, 1)	0.446	1.5 (1.03, 2.2)	0.036
WBC, 10^9^/L	0.99 (0.98, 1.02)	0.801	1.19 (0.78, 1.82)	0.43
Hemoglobin, g/dL	0.89 (0.84, 0.95)	<0.001	1.9 (1.12, 3.22)	0.018
platelets, 10^9^/L	0.99 (0.99, 0.99)	<0.001	1.21 (0.82, 1.79)	0.333
Sodium, mmol/L	1.05 (1.03, 1.07)	<0.001	1.09 (1.06~1.11)	<0.001
Potassium, mmol/L	1.18 (1.03, 1.34)	0.014	1 (0.99, 1)	0.601
Chloride, mmol/L	1.01 (0.99, 1.04)	0.171	1.03 (1.02, 1.05)	<0.001
BUN, mg/dL	1.02 (1.02, 1.03)	<0.001	0.92 (0.85, 0.98)	0.017
Creatinine, mg/dL	1.27 (1.17, 1.38)	<0.001	0.99 (0.99, 0.99)	0.022
Bicarbonate, mmoL/L	0.99 (0.95, 1.03)	0.541	1.09 (1.06, 1.11)	<0.001
Aniongap, mmoL/L	1.07 (1.04, 1.11)	<0.001	1.25 (1.07, 1.46)	0.005
INR	2.1 (1.56, 2.83)	<0.001	1.07 (1.04, 1.09)	<0.001
PT,s	1.01 (1.00, 1.01)	0.011	1.02 (1.01, 1.02)	<0.001
APTT, s	0.99 (0.99, 1.00)	0.864	1.22 (1.12, 1.32)	<0.001
GCS	0.93 (0.9, 0.98)	0.002	0.93 (0.88, 0.97)	0.002
SAPS II	1.04 (1.03, 1.05)	<0.001	1.09 (1.06, 1.13)	<0.001
APS III	1.01 (1.01, 1.02)	<0.001	2.24 (1.54, 3.27)	<0.001

SAH, subarachnoid hemorrhage; SBP, systolic blood pressure; DBP, diastolic blood pressure; MBP, mean blood pressure; RR, respiratory rate; HR, heart rate; SpO2, percutaneous oxygen saturation; SAPS II, simplified acute physiology score II; GCS, Glasgow Coma Score; Chronic pulmonary disease, chronic obstructive pulmonary disease; WBC, white blood cell; BUN, blood urea nitrogen.

Using a Cox proportional risk model, Table 4 shows uncorrected and adjusted assessments of serum sodium levels with all-cause mortality in patients with non-traumatic SAH. Serum sodium, when used as a continuous variable, was associated with an elevated risk of ICU and hospital all-cause mortality [HR: 1.05 (95% CI: 1.03–1.08) and HR: 1.09 (95% CI: 1.06–1.12)]. With each 1-unit rise in serum sodium, non-traumatic SAH ICU and hospital all-cause mortality rose. Serum sodium levels were evaluated as a categorical variable for ICU and hospital all-cause mortality. Normal blood sodium levels (135–145 mmol/L) were used as the reference. In the crude model, high serum sodium levels were associated with increased risk of ICU [HR: 1.65 (95% CI: 1.26–2.17)] and hospital all-cause mortality [HR: 2.88 (95% CI: 2.07–4.02)], respectively. In Model I, adjusting for age, sex, and race was associated with increased ICU risk [HR: 1.75 (95% CI: 1.33–2.31)] and increased hospital all-cause mortality [HR: 2.96 (95% CI: 2.12–4.13)], respectively. In addition, in Model II, adjusting for age, sex, race, SBP, DBP, RR, heart rate, GCS, diabetes, sepsis, renal failure, chronic lung disease, blood pressure elevators, WBC, platelets, hemoglobin, glucose, BUN, creatinine, anion gap, SAPS II, APS III with the normal serum sodium group as the reference, and higher serum sodium was still associated with increased ICU [HR: 1.47 (95% CI: 1.07–2.01)] and an increase in hospital all-cause mortality [HR: 2.26 (95% CI: 1.54–3.32)] were significantly associated. Regarding sensitivity analysis, serum sodium levels were evaluated for ICU and hospital all-cause mortality as a continuous and categorical variable, producing consistent results.

**Table 4:** Multivariate cox analysis between serum sodium level and mortality.

**Variable**	**Crude**		**Model I**		**Model II**	
	**HR (95% CI)**	***P*** **-value**	**HR (95% CI)**	***P*** **-value**	**HR (95% CI)**	***P*** **-value**
**ICU all-cause mortality**						
Sodium 1, mmol/L	1.05 (1.03–1.07)	<0.001	1.06 (1.04–1.09)	<0.001	1.05 (1.03–1.08)	<0.001
**Sodium tertials, mmol/L**						
<135	0.76 (0.41–1.38)	0.364	0.77 (0.42–1.41)	0.394	0.73 (0.39–1.36)	0.32
135–145	1 (Ref)		1 (Ref)		1 (Ref)	
≥145	1.65 (1.26–2.17)	<0.001	1.75 (1.33–2.31)	<0.001	1.47 (1.07–2.01)	0.017
**Hospital all-cause mortality**						
Sodium 1, mmol/L	1.09 (1.06–1.11)	<0.001	1.1 (1.07–1.12)	<0.001	1.09 (1.06–1.12)	<0.001
**Sodium tertials, mmol/L**						
<135	1.22 (0.59–2.5)	0.593	1.12 (0.54–2.3)	0.76	1.14 (0.53–2.42)	0.743
135-145	1 (Ref)		1 (Ref)		1 (Ref)	
≥145	2.88 (2.07–4.02)	<0.001	2.96 (2.12–4.13)	<0.001	2.26 (1.54–3.32)	<0.001

Crude model: adjusted for none.

Model I: adjusted for age, sex, and ethnicity.

Model II: adjusted for Model I+SBP, DBP, respiratory rate, heart rate, GCS, SAPS II, APS III, diabetes, renal failure, chronic pulmonary disease, WBC, platelet, hemoglobin, glucose, BUN, creatinine, bicarbonate, and chloride.

**Figure 3:**
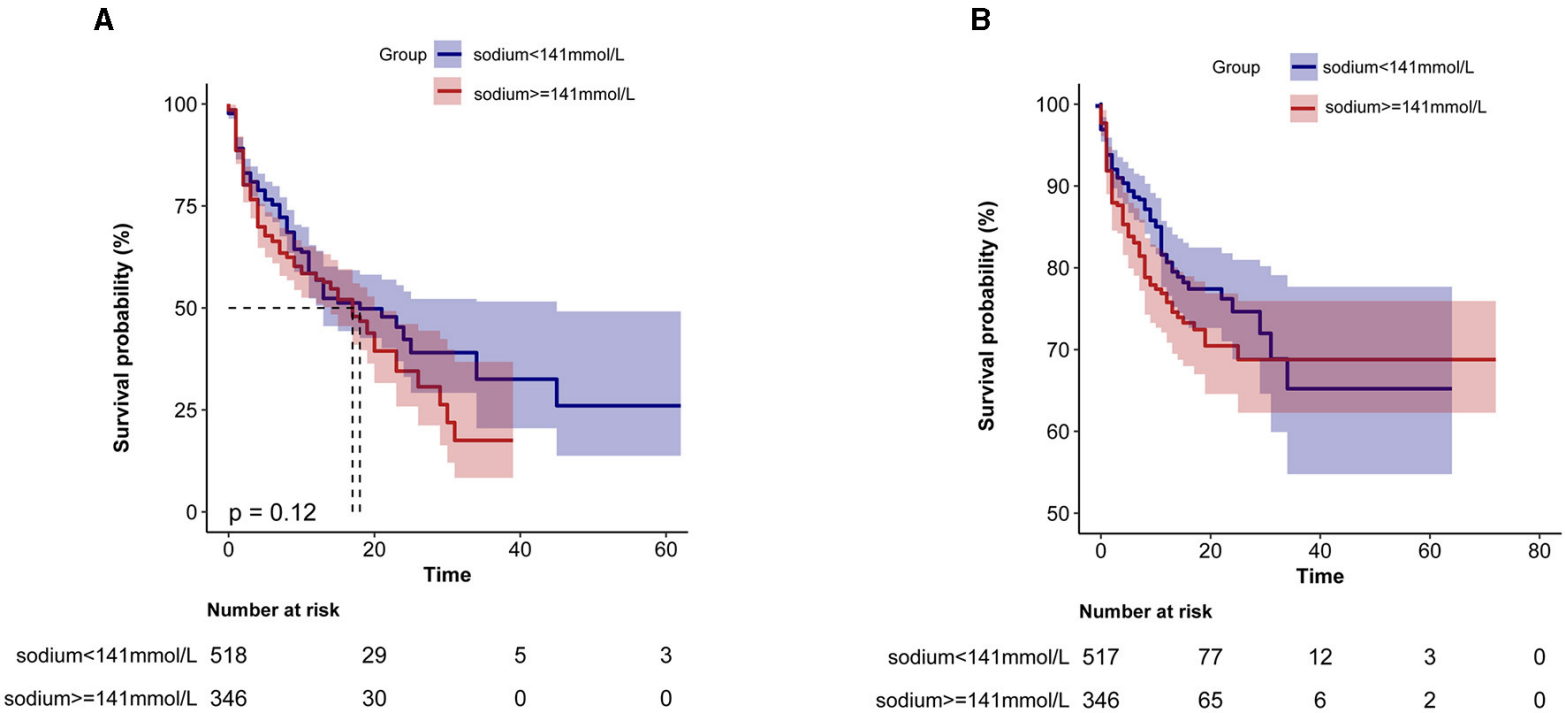
Kaplan-Meier survival curve based on serum sodium ions in critically ill patients with non-traumatic SAH. **(A)** ICU all-cause mortality; **(B)** Hospital all-cause mortality. *x*-axis: survival time (days). *y*-axis: survival probability.

Furthermore, as shown in Figure 3, KM survival curves revealed that patients with high serum sodium levels (≥141 mmol/L) on admission were related to decreased ICU risk and in-hospital survival (*p* < 0.0001).

### 3.3. Subgroup analysis

**Figure 4:**
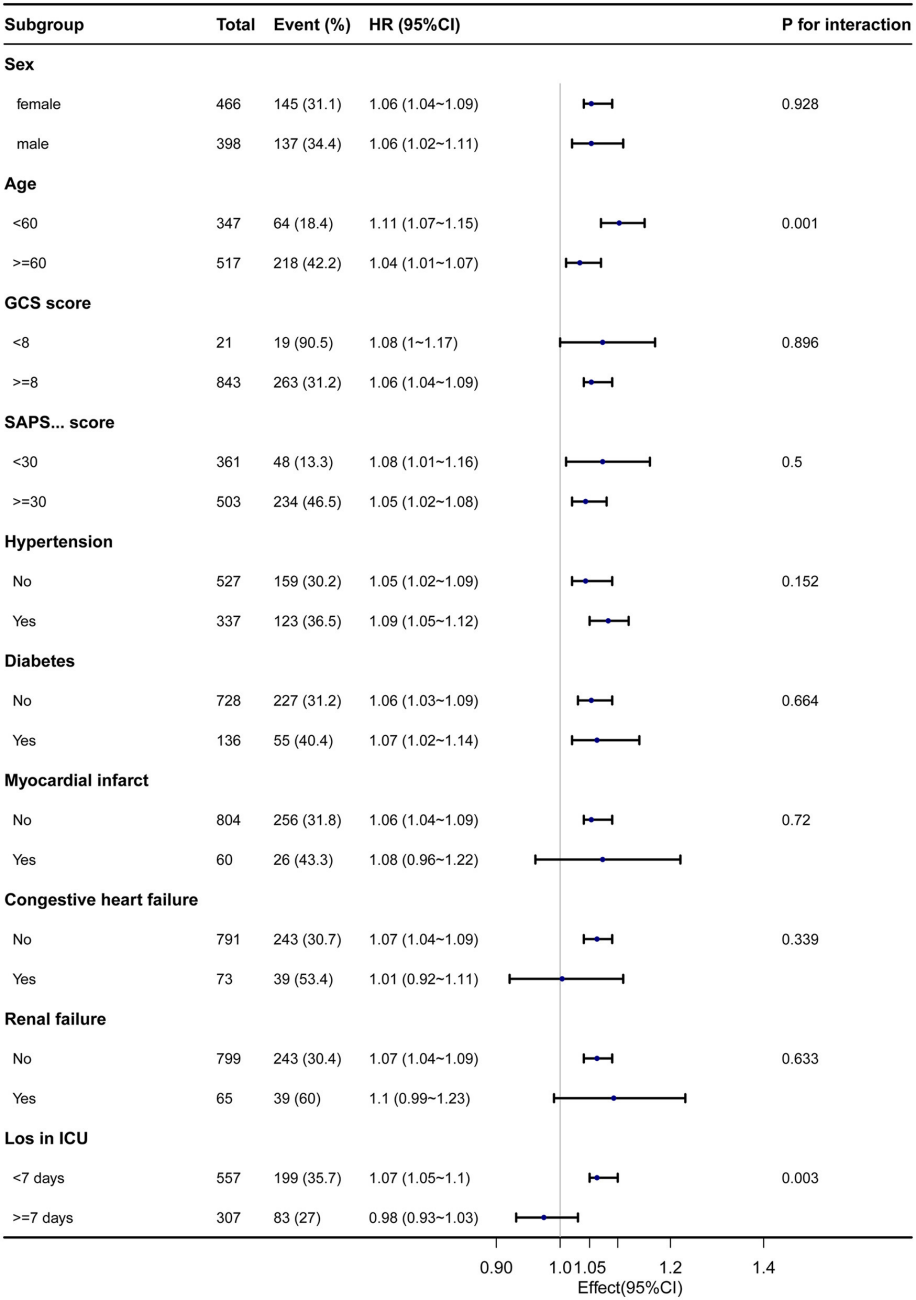
The relationship between serum sodium and in-hospital mortality in subgroup analysis.

In the subgroup analysis between the categorical variables of serum sodium within 24 h of admission and hospital death, as shown in Figure 4, we found that only age and ICU length of stay revealed differences in the subgroup analysis between the categorical variables of serum sodium and hospital death, *P* = 0.001, 0.003, respectively). Patients with older age and those admitted to ICU <7 days had a worse prognosis.

## 4. Discussion

Our current retrospective cohort study found that serum sodium levels at ICU admission were independently associated with in-hospital mortality and the risk of ICU death in critically ill patients with non-traumatic SAH, both as continuous and categorical variables. Furthermore, the relationship between serum sodium at admission and mortality was J-shaped, indicating a non-linear association. The inflection point analysis revealed that when serum sodium concentration exceeded 141 mmol/L, there was a significant positive correlation with mortality rate. However, this association was not observed when serum sodium concentration was below 141 mmol/L. After controlling for possible confounders, these correlations remained. When serum sodium was considered as a categorical variable, ICU mortality and in-hospital mortality were significantly higher for patients with serum sodium levels ≥ 145 mmol/L. On the other hand, when serum sodium was <135 mmol/L, there was no significant difference in ICU mortality and in-hospital mortality compared to the group with serum sodium levels ranging from 135 to 145 mmol/L.

Sodium anomalies are common in critically ill patients throughout their ICU admission (12). It is widely established that sodium is primarily metabolized by the kidneys and that sodium metabolism control is complicated, including numerous neurohumoral regulatory systems, including the renin-angiotensin-aldosterone system (13). In clinical work, serum sodium measurement is very relevant and simple to measure, which is the reason why all patients admitted to the ICU need to measure it. As a result, the relationship between serum sodium levels and clinical prognosis in critical care has been the subject of several investigations. According to a recent study that used data from the MIMIC database, both hyponatremia and hypernatremia were associated with increased 1- and 3-year mortality in critically ill patients with concomitant COPD (14). Patients with congestive heart failure showed a U-shaped relationship between serum sodium levels and all-cause mortality, and congestive heart failure mortality was enhanced by both hyponatremia and hypernatremia (15). Previous research has shown that sodium metabolism disorders are common in SAH patients, and hyponatremia after SAH is primarily seen in cerebral salt-wasting syndrome and antidiuretic hormone hypersecretion syndrome, whereas hypernatremia is also common in critically ill SAH patients, but the mechanisms are currently unknown (16, 17). However, in the current study reports, there is no consensus on whether disorders of blood sodium metabolism lead to a poorer prognosis in SAH patients. According to the results of our study, admission serum sodium was associated with ICU mortality and in-hospital mortality type J in SAH. There was no association between serum sodium values below 141 mmol/L and in-hospital mortality. This is mostly consistent with prior similar research (18, 19). After controlling for covariates, serum sodium, as a categorical variable, was 1.47 and 2.26 times greater than the risk of ICU and hospital all-cause mortality in patients with ≥145 mmol/L than in those with normal serum sodium levels. Furthermore, recent systematic reviews and meta-analyses have demonstrated that serum sodium is an accurate tool for predicting the prognosis of critically ill patients (15, 20, 21). According to research, hypertension, diabetes, congestive heart failure, and renal failure are typical co-morbidities in individuals with non-traumatic SAH (22, 23). Despite the fact that these co-morbidities were linked to poor outcomes, our stratified and subgroup analyses had no impact on the final outcome. Only an interaction between age and period of ICU admission was discovered in subgroup analysis.

Although the exact mechanism by which elevated serum sodium levels contribute to an increased mortality rate is not yet fully understood, several potential explanations for this phenomenon have been proposed. One possible medical factor contributing to hypernatremia in ICU patients is the excessive use of hypertonic solutions coupled with inadequate water supplementation. In a large retrospective study conducted across multiple centers, Oude Lansink-Hartgring et al. observed a shift in the occurrence of hypernatremia over the past two decades, transitioning from hyponatremia to hypernatremia. This change may be attributed to increased usage of sodium-containing infusions, hydrocortisone, and diuretics (24). However, Harada et al. reported no association between changes in serum sodium levels and cerebral vasospasm or subsequent outcomes in patients with non-traumatic subarachnoid hemorrhage (aSAH) (25). On the other hand, Jin et al. discovered a J-shaped relationship between serum sodium levels upon ICU admission, the minimum sodium values during the ICU stay, and in-hospital mortality among non-traumatic SAH patients. Sodium fluctuations exceeding 8.5 mmol/L were independently linked to in-hospital mortality (19), which aligns with our findings. Nevertheless, the precise mechanisms through which serum sodium levels impact mortality rates in aSAH patients remain unknown. Experimental studies have suggested that sustained elevation of local pressure caused by subarachnoid blood clots and compression of basal cisterns directly impairs the hypothalamic nuclei, particularly the supraoptic and paraventricular nuclei. This impairment leads to disrupted secretion of arginine vasopressin (AVP) and subsequent central diabetes insipidus, resulting in electrolyte imbalances, osmotic fluctuations, disturbance of the internal environment, and ultimately influencing mortality rates in experimental animals (26, 27). Furthermore, animal experiments have confirmed a significant reduction in AVP mRNA in the hypothalamus on the 1st and 2nd days following cortical impact in a rat model, providing further support for the aforementioned hypothesis (28).

However, this study has several noteworthy limitations. First, in the MIMIC-IV database, we were unable to obtain covariates such as delayed cerebral ischemia, hydrocephalus, and intracranial hypertension. Additionally, other residual confounders may potentially exist, as is the case with all retrospective analyses. Due to the large amount of missing data, although we used interpolation, it was still difficult to avoid bias. In addition, because of the limitations of the MIMIC database, we were unable to assess blood glucose levels, which induce hyponatremia. Second, it was a single-center retrospective research study, which is constrained by the nature of its design. Third, the outcomes of patients with non-traumatic SAH and hyponatremia might be greatly impacted by the lack of specific information on hypertonic treatment, including dosage and exact timing. The absence of this information hampered our knowledge of the various causes of serum sodium changes. Fourth, we were only able to demonstrate a connection between serum sodium levels and the overall mortality rate and not a direct causative link. However, the link between high serum sodium levels and the overall mortality rate was convincingly confirmed.

## 5. Conclusion

In patients with non-traumatic SAH, we observed a J-shaped correlation between serum sodium levels at admission and ICU and hospital mortality rates. Additionally, admission sodium levels > 141 mmol/L were significantly associated with an increased risk of death. Our findings emphasize the importance of appropriately controlling serum sodium levels to prevent hypernatremia and reduce mortality. However, prospective studies are needed to validate these results.

## Data availability statement

The data analyzed in this study was obtained from the Medical Information Mart for Intensive Care IV (MIMIC-IV) database, the following licenses/restrictions apply: to access the files, users must be credentialed users, complete the required training (CITI Data or Specimens Only Research) and sign the data use agreement for the project. Requests to access these datasets should be directed to PhysioNet, https://physionet.org/, doi: 10.13026/6mm1-ek67.

## Ethics statement

Ethical review and approval was not required for the study on human participants in accordance with the local legislation and institutional requirements. Written informed consent from the patients/participants or patients/participants' legal guardian/next of kin was not required to participate in this study in accordance with the national legislation and the institutional requirements.

## Author contributions

JLiu and JuZ designed this study, analyzed the data, and wrote the manuscript. JLi, LW, QZ, YW, and JiZ reviewed, interpreted, and checked clinical data. All authors contributed to the article and approved the submitted version.
